# Automated echolocation classifiers vary in accuracy for northeastern U.S. bat species

**DOI:** 10.1371/journal.pone.0300664

**Published:** 2024-06-03

**Authors:** Donald I. Solick, Bradley H. Hopp, John Chenger, Christian M. Newman

**Affiliations:** 1 Electric Power Research Institute, Palo Alto, California, United States of America; 2 Vesper Bat Detection Services, Fort Collins, Colorado, United States of America; 3 Bat Conservation and Management, Carlisle, Pennsylvania, United States of America; Bowling Green State University, UNITED STATES

## Abstract

Acoustic surveys of bat echolocation calls are an important management tool for determining presence and probable absence of threatened and endangered bat species. In the northeastern United States, software programs such as Bat Call Identification (BCID), Kaleidoscope Pro (KPro), and Sonobat can automatically classify ultrasonic detector sound files, yet the programs’ accuracy in correctly classifying calls to species has not been independently assessed. We used 1,500 full-spectrum reference calls with known identities for nine northeastern United States bat species to test the accuracy of these programs using calculations of Positive Predictive Value (PPV), Negative Predictive Value (NPV), Sensitivity (SN), Specificity (SP), Overall Accuracy, and No Information Rate. We found that BCID performed less accurately than other programs, likely because it only operates on zero-crossing data and may be less accurate for recordings converted from full-spectrum to zero-crossing. NPV and SP values were high across all species categories for SonoBat and KPro, indicating these programs’ success at avoiding false positives. However, PPV and SN values were relatively low, particularly for individual *Myotis* species, indicating these programs are prone to false negatives. SonoBat and KPro performed better when distinguishing *Myotis* species from non-*Myotis* species. We expect less accuracy from these programs for acoustic recordings collected under normal working conditions, and caution that a bat acoustic expert should verify automatically classified files when making species-specific regulatory or conservation decisions.

## Introduction

Identifying bat echolocation calls to species is increasingly important for monitoring North American bat populations. Traditional census methods to find and capture bats [e.g., 1,2] have become extremely difficult and labor intensive due to White Nose Syndrome (WNS), a disease caused by an invasive, pathogenic fungus that has decimated some bat populations [3,4]. For example, population declines due to WNS are responsible for uplisting the northern long-eared bat (*Myotis septentrionalis*) from threatened to endangered under the Endangered Species Act in 2022, and for the potential listing of the tricolored bat (*Perimyotis subflavus*) and little brown bat (*M*. *lucifugus*) in 2024 [5–8]. In addition, the hoary bat (*Lasiurus cinereus*) may be at risk of population decline due to wind energy development [8,9]. The primary method to characterize hoary bat activity at wind energy facilities requires acoustic monitoring.

Passive acoustic monitoring has become the preferred practice for bat species occurrence and activity surveys, and often results in higher detections of rare species than traditional capture techniques [10]. Acoustic monitoring is non-invasive, and can sample a much broader area over long periods of time. Researchers typically deploy detectors with ultrasonic microphones in areas of interest and record the echolocation calls of free-flying bats to determine the probable presence or absence of threatened and endangered species [11] or to monitor population trends [12]. A standard practice for areas under consideration for wind energy development is to conduct three to twelve months of acoustic monitoring for bat collision risk [13,14]. The value of these pre-construction surveys has recently been questioned [15,16], but acoustic monitoring may still play a role in detection-based operational minimization (a.k.a., “smart curtailment”) in reducing bat mortality at wind energy facilities [17,18].

Acoustic surveys typically yield large numbers of recordings, and manual classification of echolocation calls is subjective, time-consuming, and requires expertise [19]. To make acoustic species identification more accessible, objective, and manageable, Bat Call Identification (BCID), Kaleidoscope Pro (KPro), and SonoBat software programs were developed to perform automated classification of echolocation calls to species. These programs claim high accuracy of correct classifications, up to 100% for many species [20–22], but acknowledge that accurate species classification depends on a multitude of factors (e.g., recording quality, bat behavior, presence of conspecifics, etc. [23]), and that any final conclusions regarding species presence require confirmation by a qualified biologist. However, these classifiers’ ease of use and reported accuracy can lead to undue confidence in their results, causing several publications to caution against accepting classifier results *de facto* [24,25]. Software classifications can impact management decisions, so several studies have examined inter-classifier agreement to indirectly assess program accuracy. Pairwise agreements for the classifications of individual files have been uniformly low. Overall agreement between KPro and EchoClass was 35% for *M*. *septentrionalis* in Michigan [26], 38% for BCID and EchoClass for eight species in Ohio [27], and averaged approximately 40% across four programs (KPro, EchoClass, BCID, and SonoBat) for seven species in Nebraska [28]. Nocera et al. (2019) [29] found low interprogram agreement between KPro, EchoClass, and BCID, as measured by Cohen’s Kappa (0.2–0.6), for nine species in New York. However, these studies assessed classifiers using recordings of free-flying bats. As such, researchers did not have information on true species identifications, precluding calculations of direct program accuracy.

Species identification of bat echolocation calls is difficult. Bat echolocation is primarily used for navigation and foraging in the dark, so echolocation calls are therefore simple, variable, and context-dependent compared to the relatively complex, discernable, and species-specific songs of birds and frogs [30,31; but see 32]. When rendered as a spectrogram, the frequency and duration of echolocation calls are highly variable within species [33,34], and these parameters have considerable overlap among species that forage in the same habitat, hunt the same prey, and/or have common ancestry [31]. For example, the federally endangered *M*. *septentrionalis* and *M*. *sodalis* are routinely confused for other species in the northeastern United States (U.S.), particularly with the relatively common eastern red bat (*Lasiurus borealis*; [27,29,35,36]). The U.S. Fish and Wildlife Service (USFWS) requires acoustic surveys that determine nightly probable presence or absence for these protected species to use approved automated classifier programs that calculate a maximum likelihood estimate (MLE). MLE values compare the number of files classified as each identified species to the known misclassification rates of those species in the underlying classifier algorithm [37]. If presence is likely for a given night (MLE p ≤ 0.05), then either all recorded calls from that night must be manually reviewed by a bat acoustic expert to verify presence, or follow-up capture surveys must be completed to verify presence. If presence of *M*. *septentrionalis* or *M*. *sodalis* is considered unlikely (MLE p > 0.05), then no further surveys are required [11]. Because false positives and false negatives could have important consequences for management and conservation decisions for these and other bat species, an independent assessment of the accuracy of automated classification programs is warranted.

To make informed decisions on bat conservation and management in North America, it is crucial to understand the differences in species identification accuracy across automated classification programs. We tested the accuracy of commercially available automated classification programs (BCID, KPro, SonoBat) using a dataset of known echolocation calls for nine northeastern U.S. species. Based on the low pairwise agreements among these programs in other studies [26–28], we hypothesized that the programs’ classification accuracy would differ and that classification will be more accurate for species that produce more distinct echolocation calls compared to species that produce calls that are more ambiguous [33].

## Methods

### Data collection

We collected 1,940 reference call recordings from seven researchers for nine bat species that occur in the northeastern U.S.: big brown bat (*Eptesicus fuscus*), eastern red bat (*Lasiurus borealis*), hoary bat (*L*. *cinereus*), silver-haired bat (*Lasionycteris noctivagans*), eastern small-footed bat (*Myotis leibii*), little brown bat (*M*. *lucifugus*), northern long-eared bat (*M*. *septentrionalis*), Indiana bat (*M*. *sodalis*), and tricolored bat (*Perimyotis subflavus*). This species assemblage included endangered U.S. species of interest, and consisted of the nine species found in New York, Vermont, Connecticut, and Massachusetts.

The methods and materials used to collect reference calls varied among researchers, but they made all recordings using full-spectrum detectors (e.g., Pettersson D240x and D500x, Wildlife Acoustics SM2BAT, Binary Acoustic Technology AR125) to record free-flying individuals that had either been a) positively identified to species in the hand prior to release or b) positively identified in flight (for visually distinct species due to coloration; e.g., *L*. *boreali*s) with illumination by a high-powered spotlight. In general, one or more observers visually tracked flying bats with detectors. Most recordings of captured bats started immediately upon release and ended when the bat was lost from sight, usually no more than 20 seconds after release but often much less. In some instances, observers illuminated the target bat during recording. Recording locations varied from releases under canopy to open lawns surrounded by multiple observers. We digitally recorded echolocation calls to memory cards, and later downloaded the calls to computers and where we labeled them by species, location, time, and other pertinent recording information. We visually inspected all reference calls, and only accepted recordings that contained a minimum of five search phase pulses [38]. We excluded recordings of bats that were not free-flying (e.g., zip-lined bats, or bats exiting a roost), that had low sound quality, and that contained calls by multiple individuals or species. We also excluded recordings that had been used in the training datasets for the software programs we were testing, with the exception of six *M*. *leibii* calls and 24 *M*. *sodalis* calls. We retained these recordings for analyses to increase the sample size of these species for all three programs.

We accepted 1,120 files to analyze (S3 Table in S2 File). We used an additional 380 recordings that did not contain bat signals to represent noise recordings (e.g., rustling vegetation, insects, rain), for a total of 1,500 recordings used in analyses. We collected most reference calls (84%) at locations throughout the eastern U.S., and we obtained additional calls for six of the species from other researchers (S3 Table in S2 File). Approximately 16% of calls came from outside the northeastern U.S., most of which were for *L*. *noctivagans*, *L*. *borealis*, and *L*. *cinereus*. The extent of geographic variation in echolocation calls is unknown for most species of bats, but is believed to be minor and primarily driven by differences in temperature and humidity across a species’ range [31]. SonoBat and KPro training datasets utilize reference calls from across the full species’ range, so the echolocation call files’ geographic origin presumably has a negligible effect on program performance.

### Software and settings

We used the most recent version of SonoBat (30.0), and the most recent USFWS-approved versions of KPro (5.4.7) and BCID (2.8b) for automated classification of *M*. *septentrionalis* and *M*. *sodalis* [14], as the programs we compared in our analysis. The USFWS has approved no version of SonoBat because there is no available dataset of full-spectrum reference calls to test the program’s accuracy. SonoBat only processes full-spectrum WAV recordings, and classifies files based on analysis of more than 100 call parameters with a hierarchical expert decision system based on a library of 45,000 known bat passes for eastern North America (https://sonobat.com/sonobat_classification). We used the NY–PA suite within the SonoBat classifier for northeast North America. This suite includes Rafinesque’s big-eared bat (*Corynorhinus rafinesquii*) along with the other nine bat species we considered in our analysis. SonoBat also uses a classification category called “LUSO” for calls that are ambiguous between *M*. *lucifugus* and *M*. *sodalis*. We maintained this category in our analysis except when grouping all *Myotis* species calls (MYOTIS), in which case we added LUSO to MYOTIS. We used default settings for Sequence Decision Threshold (0.90), Acceptable Call Quality (0.6), and Maximum Number of Calls to Consider per File [32].

Kaleidoscope Pro utilizes Hidden Markov Models to recognize temporal patterns and assign classifications using Fisher scores, clustering, and pairwise algorithms [39]. We used the New York set of species, limited to the nine species in this study, in the Bats of North America 5.4.0 classifier. KPro operates on zero-crossing data extracted from full-spectrum recordings within the program. During analysis (and conversion to zero-crossing recordings for BCID; see below), we used default settings with the following modifications: we selected Advanced Signal Processing, Minimum and Maximum Frequency Range (8–120 kHz), Minimum and Maximum Length of Detected Pulses (2–500 ms), and Maximum Inter-syllable Gap (500 ms). We also selected 5 as the Minimum Number of Pulses and “-1 (More Sensitive)” as the Sensitivity setting, as per USFWS guidance [14].

BCID is a weighted classification tree analysis that only operates on zero-crossing data. Our attempts to generate zero-crossing recordings using default settings for BCID’s built-in WavToZero function were unsuccessful, so we used KPro-generated zero-crossing recordings as described above in this analysis. We initially disabled Advanced Signal Processing during conversion, but BCID performed better on recordings with Advanced Signal Processing enabled so we used those converted recordings instead. Kaleidoscope Pro could not convert two NOISE recordings to zero-crossing format; as such, we could not use these two recordings in our analysis of BCID. We used the New York set of species in the classifier, which included just the nine species of interest in this study. We used the USFWS-approved settings for the built-in BCID filter and set Minimum Discriminant Probability to 0.35 [14].

We considered including a fourth program in our study. This program, EchoClass, also operates on zero-crossing data. However, the version we downloaded (3.1; May 2015) did not process our data due to a flaw in the program, and we could not resolve this before we concluded this study (E. Britzke, personal communication).

### Data analysis

We generated confusion matrices for each species by each program using the R package “caret” version 6.0–94 [40] (S1 File). A confusion matrix is a table that compares known values with predicted values to derive correct and incorrect classifications. We used the known species of each file as the reference, and used the output classifications of the three programs as predictions. All three programs in this study had functionality to indicate a bat was present even if it could not be identified to species. We used the term “UNKW” to classify these predictions and counted UNKW calls as misclassifications in our metrics. In addition to examining each species individually, we grouped all four *Myotis* species into a new category, MYOTIS, to determine classification rates for this grouping versus other northeastern species.

We calculated sensitivity (SN), negative predictive value (NPV), specificity (SP), positive predictive value (PPV), overall accuracy (AC), and the no information rate (NIR) for each classification category. We considered accuracy metrics that exceeded 0.80 for a species to represent good performance by a program.

We defined “Target Species” as the individual species we were examining, and grouped all other species (and NOISE) into the category “OTHER.” PPV is the proportion of correct Target Species classifications (true positives) to the total number of true positive and false positive classifications. In this study, NPV was the proportion of correct OTHER classifications (true negatives) to the total number of true negative and false negative classifications. PPV and NPV estimated the probability a positive or negative classification was true based on the prevalence of the Target Species in the dataset. Special care must be given when interpreting NPV. If there are many OTHER files compared to Target Files (as was the case for most of our species), then a high NPV could be achieved even if all Target Species are incorrectly classified as OTHER (S1 File).

Sensitivity was the proportion of correct Target Species classifications (true positives) to the total number of true positive and false negative classifications. Specificity was the proportion of correct OTHER classifications (true negatives) to the total number of true negative and false positive classifications (correct and incorrect). Higher SN and SP indicated better performance at avoiding false negatives and positives, respectively.

Overall Accuracy was calculated as the total number of correct Target Species and OTHER classifications divided by the total number of recordings. We computed AC along with a 95% confidence interval using a Clopper and Pearson exact binomial test [41] to compare AC with NIR. The NIR was how often a classifier would be correct if it always identified a file as the most common category (OTHER, in this study). To be significant, AC needed to exceed NIR. We used the binom.test() function in the base R package “stats,” repeating the test for each test category (nine species, MYOTIS, and Noise) yielding 11 separate p-values.

## Results

**Fig 1:**
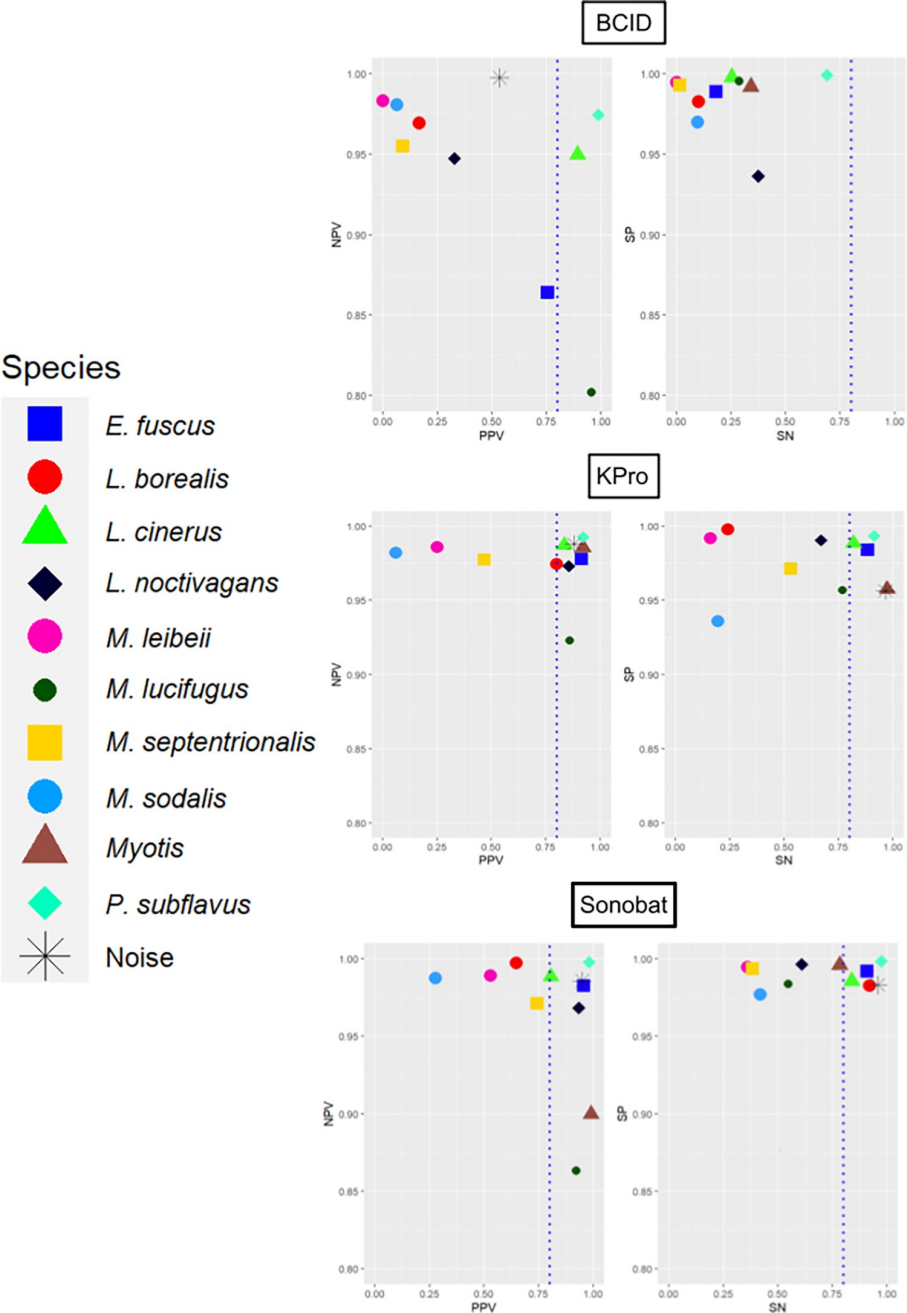
Comparisons of Negative Predictive Value (NPV) and Positive Predictive Value (PPV; left-most graphs) and comparisons of Specificity (SP) and Sensitivity (SN; right-most graphs) for automated classifications of 1,500 reference calls for nine northeastern U.S. bat species by BCID, KPro, and Sonobat programs. Symbols to the right of the dashed line indicate classification categories with accuracy metrics > 0.80.

BCID did not reach our 0.80 threshold of acceptable accuracy for every metric in any classification category (Table 1). Of the 11 classification categories, BCID performed best at classifying *P*. *subflavus*, *L*. *cinereus*, and *M*. *lucifugus*, although SN was 0.68 for *P*. *subflavus* and < 0.30 for *L*. *cinereus* and *M*. *lucifugus* (Fig 1 and Table 1). KPro and SonoBat exceeded 0.80 across all five metrics for five and four classification categories, respectively (Table 1). KPro performed best for classifying *E*. *fuscus*, *L*. *cinereus*, *P*. *subflavus*, MYOTIS, and NOISE, while SonoBat performed best for classifying *E*. *fuscus*, *L*. *cinereus*, *P*. *subflavus*, and NOISE (Fig 1 and Table 1). None of the programs performed well for classifying *M*. *leibii*, *M*. *septentrionalis*, or *M*. *sodalis*, with SN and PPV less than 0.80 and non-significant AC (except for SonoBat, which had significant AC for *M*. *septentrionalis*; Fig 1 and Table 1).

**Table 1:** Summary of accuracy metrics across BCID, KPro, and Sonobat automated classification programs for nine northeastern U.S. bat species.

	BCID						KPro						Sonobat					
	SN	PPV	NPV	SP	AC	pval	SN	PPV	NPV	SP	AC	pval	SN	PPV	NPV	SP	AC	pval
***E*. *fuscus***	0.17	0.75	0.86	0.98	0.85	**0.02**	0.88	0.91	0.97	0.98	0.96	**0.00**	0.90	0.95	0.98	0.99	0.97	**0.00**
***L*. *borealis***	0.1	0.16	0.96	0.98	0.95	0.99	0.24	0.8	0.97	0.99	0.97	0.1	0.92	0.64	0.99	0.98	0.98	**0.00**
***L*. *cinereus***	0.25	0.89	0.94	0.99	0.94	**0.01**	0.81	0.83	0.98	0.98	0.97	**0.00**	0.83	0.8	0.98	0.98	0.97	**0.00**
***L*. *noctivagans***	0.37	0.32	0.94	0.93	0.89	0.99	0.66	0.85	0.97	0.99	0.96	**0.00**	0.6	0.93	0.96	0.99	0.96	**0.00**
***M*. *leibii***	0	0	0.98	0.99	0.97	0.95	0.16	0.25	0.98	0.99	0.97	0.95	0.36	0.52	0.98	0.99	0.98	0.47
***M*. *lucifugus***	0.28	0.95	0.8	0.99	0.81	**0.00**	0.76	0.86	0.92	0.95	0.9	**0.00**	0.54	0.92	0.86	0.98	0.87	**0.00**
***M*. *septentrionalis***	0.01	0.09	0.95	0.99	0.94	0.87	0.52	0.46	0.97	0.97	0.95	0.75	0.38	0.74	0.97	0.99	0.96	0.01
***M*. *sodalis***	0.09	0.06	0.98	0.97	0.95	0.99	0.19	0.05	0.98	0.93	0.92	1	0.41	0.27	0.98	0.97	0.96	0.99
**MYOTIS**	0.34	0.95	0.74	0.99	0.77	**0.00**	0.97	0.92	0.98	0.95	0.96	**0.00**	0.78	0.99	0.89	0.99	0.92	**0.00**
***P*. *subflavus***	0.68	0.98	0.97	0.99	0.97	**0.00**	0.91	0.92	0.99	0.99	0.98	**0.00**	0.97	0.98	0.99	0.99	0.99	**0.00**
**NOISE**	0.99	0.53	0.99	0.71	0.78	**0.00**	0.96	0.88	0.98	0.95	0.95	**0.00**	0.95	0.94	0.98	0.98	0.97	**0.00**

SN = Sensitivity, NPV = Negative Predictive Value, SP = Specificity, PPV = Positive Predictive Value, AC = Accuracy, pval = p-value for No Information Rate binomial test with Accuracy. Green indicates values greater than 0.80. Bold p-values indicate significance. MYOTIS = combined files for *M*. *leibii*, *M*. *lucifugus*, *M*. *septentrionalis*, and *M*. *sodalis*; NOISE = non-bat. A twelfth category, “UNKW”, is not listed since it refers to when a program estimates the file contains a bat, but did not assign a species.

Across programs, measures of NPV, SP, and AC were relatively high, exceeding 0.80 for nearly all classification categories (Table 1) except for BCID classifications of MYOTIS and NOISE. Yet AC was only significantly less than the NIR for six BCID classification categories, seven KPro classification categories, and nine SonoBat classification categories (Table 1). All three programs performed relatively poorly for measures of SN and PPV. BCID only exceeded 0.80 for SN with NOISE, while KPro and SonoBat exceeded 0.80 for SN for just five of 11 classification categories (Fig 1 and Table 1). BCID exceeded a PPV of 0.80 for four classification categories, SonoBat exceeded a PPV of 0.80 for seven categories, and KPro exceeded a PPV of 0.80 for eight classification categories (Fig 1 and Table 1). BCID, KPro, and Sonobat classified 299, 7, and 115 recordings as UNKW, respectively (S1 Table in S2 File).

## Discussion

Accuracy varied among automated classification programs for the nine northeastern bat species tested in our analyses. This study was the first comprehensive assessment of accuracy for commercially available programs in the United States. Goodwin and Gillam [42] focused on pairwise agreement between different versions of KPro and SonoBat, but reported 0.32–0.70 AC and 0.0–0.84 SN for full-spectrum known-species recordings (254 for KPro, 312 for SonoBat) of seven species included in our study (*E*. *fuscus*, *L*. *noctivagans*, *L*. *borealis*, *L*. *cinereus*, *M*. *septentrionalis*, and *P*. *subflavus*). In our study, AC and SN for the same species was higher, ranging between 0.85 and 0.99, and between 0.01 and 0.97, respectively, for all three programs. Goodwin and Gillam [42] did not report the specific number of recordings used for each species, and did not indicate that their recordings were filtered for quality, so the higher accuracies we report in our study may be due to larger sample sizes and the higher quality recordings we used in our analyses.

Our results were consistent with tests of similar software using known echolocation calls in Australia and Europe [43,44]. These studies found that, while automated classification can be useful for monitoring wild bat populations, the programs’ ability to accurately identify unknown bat calls to species is severely limited and caution should be exercised when interpreting results [24,25]. While our analyses intentionally used high-quality, hand-picked echolocation calls by known species to provide a best-case scenario for automated classifiers, all three programs struggled with accuracy for some species, obtaining metrics well below what is advertised [20–22]. We expect lower accuracy for acoustic recordings collected under normal working conditions that can contain multiple bats, be highly variable in quality, and that may be recorded in different habitats or by bats exhibiting different behaviors than the reference calls used in the training libraries [45].

SonoBat and KPro were comparable in terms of accuracy, and both programs performed best at correctly classifying known calls of *E*. *fuscus*, *L*. *cinereus*, *P*. *subflavus*, and NOISE, with marginal performance for *L*. *borealis* and *L*. *noctivagans*. These species produce relatively distinct calls [33], supporting our hypothesis that accuracy would be highest for less ambiguous species. *Lasiurus cinereus* and *P*. *subflavus* are experiencing population declines and may soon be federally protected [6,46], so our results indicate that automated acoustic monitoring may be a viable conservation and management tool for these species. Users of these programs can be relatively confident in automated classifications for these species using the settings described here, but it is unknown how well these classifiers would perform on data collected outside the northeastern U.S. It is possible that accuracy would be different in areas with a smaller subset of the species used in our analyses, where bats may alter their echolocation calls in the absence of competition for acoustic space [47], and accuracy may decrease in areas of greater diversity that include species not tested here. We must also acknowledge that we used USFWS-recommended settings for BCID and KPro to optimize performance for identifying *M*. *septentrionalis* and *M*. *sodalis* [14]. Different settings for any program may affect classification rates for different species.

None of the programs reliably identified calls by individual *Myotis* species, though all three programs performed marginally well for correctly classifying *M*. *lucifugus*. The *Myotis* species tested here produce echolocation calls with overlapping frequency characteristics [33], making species discrimination difficult [48]. *Myotis septentrionalis* and *M*. *sodalis* are federally endangered *Myotis* species, and a decision on the regulatory status of *M*. *lucifugus* is currently pending [49]. The inability for automated classification programs to accurately identify high quality, known calls for these species illustrates the importance of manual verification of *Myotis* species by an acoustic expert and has important implications for presence/absence surveys of protected species. Based on the USFWS guidelines, acoustic presence is established from MLE values calculated by comparing the number of recordings classified as a species on a given night with misclassification rates for each program [11,37]. With SN ranging between 0.01 and 0.52 for *M*. *septentrionalis* and *M*. *sodalis*, acoustic surveys would likely need to record many high-quality bat calls by these species on a single night in order to be considered statistically present. This can be a challenge when monitoring for rare species [50,51].

Our confusion matrices (S1 File) indicate that echolocation calls by the four *Myotis* species in this study were most frequently confused with each other, and less commonly with non-*Myotis* species. As well, the relatively low PPV values for *M*. *leibii*, *M*. *septentrionalis*, and *M*. *sodalis* for all three programs were likely driven by low sample sizes in these categories. These results motivated us to include MYOTIS as a separate classification category to determine how well the programs could distinguish calls by any of the four *Myotis* from other species. KPro performed the best at correctly distinguishing *Myotis* from other species, with values greater than 0.80 for each accuracy metric, while SonoBat performed well with a value of 0.78 for SN and > 0.80 for other metrics. With two *Myotis* species in the northeastern U.S. currently endangered, and a third possibly receiving protected status in the near future, acoustic surveys to detect any *Myotis* species in the northeastern U.S. may be a more accurate and informative approach than species-specific surveys for automatically determining presence of protected bat species in this region.

The USFWS currently approves BCID for automated classification of *M*. *septentrionalis* and *M*. *sodalis*, yet our analysis found that this program did not exceed 0.80 for any accuracy metric for any species. SN was relatively low for most classification categories, indicating that BCID is prone to false negatives. BCID was also prone to misclassifying bat calls as NOISE. Because the Master Test Library maintained by the USFWS (in collaboration with the United States Geological Survey Virginia Cooperative Fish and Wildlife Research Unit) does not currently have enough full-spectrum reference calls to adequately test SonoBat [14], we assume that USFWS tests BCID exclusively on reference calls in zero-crossing format recorded by ultrasonic recorders that are no longer manufactured or maintained (e.g., Titley Anabat SD2 detectors). BCID was likely trained on recordings by native zero-crossing detectors and may perform much better on datasets composed of similar data. However, most commercially available ultrasonic detectors now record in full-spectrum, and most BCID-analyzed files would necessarily be converted to zero-crossing by KPro or another program prior to analysis. As such, we suggest that the USFWS reconsider their approval of BCID, particularly for full-spectrum files converted to zero-crossing format, as our results suggest that these classifications have low accuracies and thus could affect management decisions regarding protected species.

In general, all three programs performed well at avoiding false positives (high SP and PPV) but were prone to false negatives (low SN). High NPV values in our study were likely a result of a high number of OTHER files relative to Target Species (S1 File) and do not necessarily indicate avoidance of false negatives. If a program classifies a recording as a particular species, our results suggest a user can have confidence that the species is actually present. However, because the programs are susceptible to false negatives, a lack of recordings from a particular species does not mean that the species is absent. This issue may be exacerbated for rare and/or acoustically ambiguous species, such as *Myotis*. For USFWS presence/absence surveys of protected species, it may be possible to ameliorate the issue of false negatives by calculating MLE [29], which can correct for false positives and false negatives by using misclassification rates. However, MLE calculations are program-specific, based on their underlying classifier algorithms and training libraries [37,45], and are therefore not standardized across the industry. Understanding the minimum number of classified calls needed by each program to accurately determine acoustic presence for each species would be valuable for assessing the limitations of these programs. For North American Bat Monitoring program surveys seeking to determine broad-scale occupancy and long-term population trends [52,53], it may be important to assess minimum survey efforts (e.g., number of survey nights) needed to effectively determine the presence or absence for different species [50,51]. Acoustic rates of occurrence may also influence decisions related to estimating whether there is risk of “take” for federal or state endangered species permits. It is also an issue for real-time classifications that may be used for species-specific curtailment of wind turbines to minimize collision risk of protected species. Understanding the potential inaccuracies of automated classifiers for different applications is important for making quality decisions.

Automated classifiers continue to improve, with versions of KPro and SonoBat becoming more conservative over time [42], but our study illustrates these programs can be highly inaccurate, particularly for ambiguous species such as *Myotis*, even when provided with high-quality recordings. All three programs have a variety of parameters that can be modified to potentially improve detection or change what type of error is seen more often. Using the results presented here, users can also select the best program suited for their species of interest and the type of error they can best tolerate. Recently developed machine learning algorithms (EchoVision [54]; NABat ML [55]) show some promise for automated classification of bat echolocation calls. However, EchoVision was trained on and is restricted to processing zero-crossing recordings, and both algorithms were trained and tested on qualitatively identified recordings rather than on recordings from known species. Until advances in signal processing demonstrate consistently high accuracy for correctly classifying bat echolocation calls collected under a wide array of field conditions, we recommend that a qualified bat acoustic analyst verify automated classifications to confirm species presence or probable absence, particularly when making species-specific conservation, regulatory, and permitting decisions.

## Supporting information

S1 File Confusion matrices. (XLSX)

S2 File Supplemental tables and figures. (DOCX)

S3 File R packages. R packages and dependencies.
(DOCX)

S4 File R script and data. (ZIP)
