# Supplementary material for: Automated echolocation classifiers vary in accuracy for northeastern U.S. bat species
Source: PLoS One. 2024 Jun 3;19(6):e0300664. doi: 10.1371/journal.pone.0300664 (PMC11146688; doi:10.1371/journal.pone.0300664)

Supplemental Tables and Figures

**Table S1:** Counts and NIR values for Sonobat, KPro, and BCID summary tables respectively


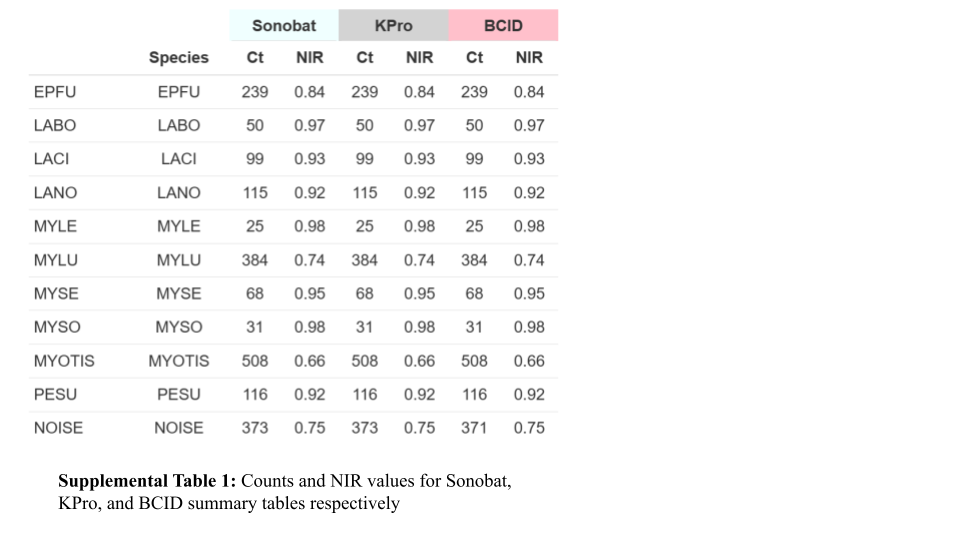


**Table S2:** Counts and hypothetical NPV values if all files are classified as OTHER. * Using a total of 1500 files, BCID only had 1498 total files. Smaller values could be obtained if any non-Target Species files were labeled as Target Species


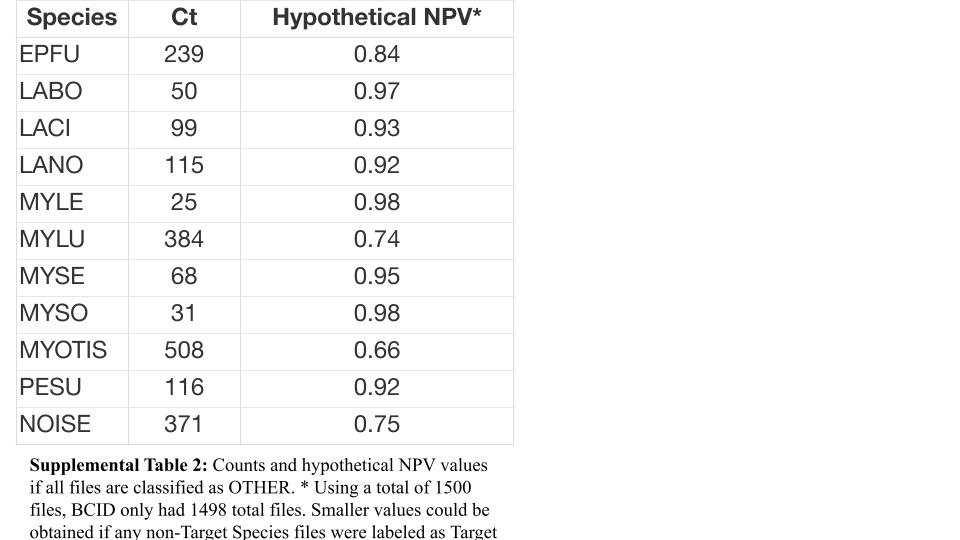


**Figure S1:** Boxplots of each metric by software. Each boxplot represents the values for each category for that metric-software combination.


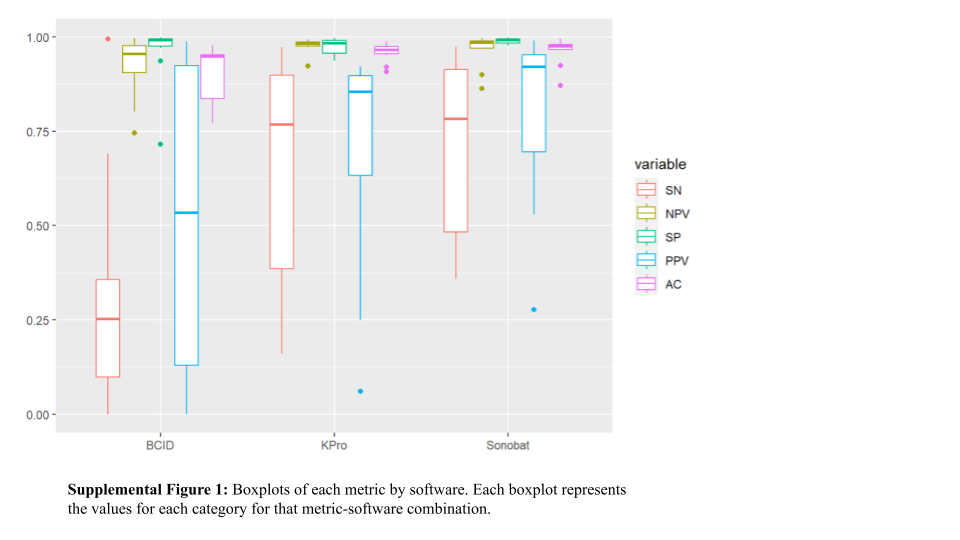

Supplement: S2 File — (DOCX) [file pone.0300664.s002.docx]
