# Supplementary material for: Automated echolocation classifiers vary in accuracy for northeastern U.S. bat species
Source: PLoS One. 2024 Jun 3;19(6):e0300664. doi: 10.1371/journal.pone.0300664 (PMC11146688; doi:10.1371/journal.pone.0300664)
Supplement: S3 File — R packages and dependencies. (DOCX) [file pone.0300664.s003.docx]

| Allaire J, Xie Y, Dervieux C, McPherson J, Luraschi J, Ushey K, et al. rmarkdown: Dynamic documents for r [Internet]. 2023. Available from: https://github.com/rstudio/rmarkdown |
| --- |
| Analytics R, Weston S. iterators: Provides iterator construct [Internet]. 2022. Available from: https://CRAN.R-project.org/package=iterators |
| Antoine Lucas DE with contributions by, Tuszynski J, Bengtsson H, Urbanek S, Frasca M, Lewis B, et al. digest: Create compact hash digests of r objects [Internet]. 2022. Available from: https://CRAN.R-project.org/package=digest |
| Auguie B. gridExtra: Miscellaneous functions for “Grid” graphics [Internet]. 2017. Available from: https://CRAN.R-project.org/package=gridExtra |
| Bates D, Eddelbuettel D. Fast  and elegant numerical linear algebra using the RcppEigen package. Journal of Statistical Software. 2013;52(5):1–24. |
| Bates D, Mächler M, Bolker B, Walker S. Fitting  linear mixed-effects models using lme4. Journal of Statistical Software. 2015;67(1):1–48. |
| Bates D, Maechler M. MatrixModels: Modelling with sparse and dense matrices [Internet]. 2022. Available from: https://CRAN.R-project.org/package=MatrixModels |
| Bates D, Mullen KM, Nash JC, Varadhan R. minqa: Derivative-free optimization algorithms by quadratic approximation [Internet]. 2022. Available from: https://CRAN.R-project.org/package=minqa |
| Bengtsson H. globals: Identify global objects in r expressions [Internet]. 2022. Available from: https://CRAN.R-project.org/package=globals |
| Bengtsson H. listenv: Environments behaving (almost) as lists [Internet]. 2022. Available from: https://CRAN.R-project.org/package=listenv |
| Bengtsson H. parallelly: Enhancing the “parallel” package [Internet]. 2023. Available from: https://CRAN.R-project.org/package=parallelly |
| Bengtsson H. progressr: An inclusive, unifying API for progress updates [Internet]. 2023. Available from: https://CRAN.R-project.org/package=progressr |
| Bryan J, Citro C, Wickham H. gargle: Utilities for working with google APIs [Internet]. 2023. Available from: https://CRAN.R-project.org/package=gargle |
| Bryan J. cellranger: Translate spreadsheet cell ranges to rows and columns [Internet]. 2016. Available from: https://CRAN.R-project.org/package=cellranger |
| Calhoun P. Exact: Unconditional exact test [Internet]. 2022. Available from: https://CRAN.R-project.org/package=Exact |
| Chang W. cachem: Cache r objects with automatic pruning [Internet]. 2023. Available from: https://CRAN.R-project.org/package=cachem |
| Chang W. fastmap: Fast data structures [Internet]. 2023. Available from: https://CRAN.R-project.org/package=fastmap |
| Chang W. R6: Encapsulated classes with reference semantics [Internet]. 2021. Available from: https://CRAN.R-project.org/package=R6 |
| Chang W. webshot: Take screenshots of web pages [Internet]. 2022. Available from: https://CRAN.R-project.org/package=webshot |
| Cheng J, Mastny T, Iannone R, Schloerke B, Sievert C. sass: Syntactically awesome style sheets (“Sass”) [Internet]. 2023. Available from: https://CRAN.R-project.org/package=sass |
| Cheng J, Sievert C, Schloerke B, Chang W, Xie Y, Allen J. htmltools: Tools for HTML [Internet]. 2023. Available from: https://CRAN.R-project.org/package=htmltools |
| Constantin AE, Patil I. ggsignif: R package for displaying significance brackets for “ggplot2”. PsyArxiv [Internet]. 2021; Available from: https://psyarxiv.com/7awm6 |
| Csárdi G, Chang W. callr: Call r from r [Internet]. 2022. Available from: https://CRAN.R-project.org/package=callr |
| Csárdi G, Chang W. processx: Execute and control system processes [Internet]. 2023. Available from: https://CRAN.R-project.org/package=processx |
| Csárdi G, FitzJohn R. progress: Terminal progress bars [Internet]. 2019. Available from: https://CRAN.R-project.org/package=progress |
| Csárdi G, Hester J, Wickham H, Chang W, Morgan M, Tenenbaum D. remotes: R package installation from remote repositories, including “GitHub” [Internet]. 2021. Available from: https://CRAN.R-project.org/package=remotes |
| Csárdi G, Müller K, Hester J. desc: Manipulate DESCRIPTION files [Internet]. 2022. Available from: https://CRAN.R-project.org/package=desc |
| Csardi G, Sorhus S. praise: Praise users [Internet]. 2015. Available from: https://CRAN.R-project.org/package=praise |
| Csárdi G. crayon: Colored terminal output [Internet]. 2022. Available from: https://CRAN.R-project.org/package=crayon |
| Csárdi G. pkgconfig: Private configuration for “R” packages [Internet]. 2019. Available from: https://CRAN.R-project.org/package=pkgconfig |
| Csardi G. prettyunits: Pretty, human readable formatting of quantities [Internet]. 2020. Available from: https://CRAN.R-project.org/package=prettyunits |
| Csardi G. rematch: Match regular expressions with a nicer “API” [Internet]. 2016. Available from: https://CRAN.R-project.org/package=rematch |
| Csárdi G. rematch2: Tidy output from regular expression matching [Internet]. 2020. Available from: https://CRAN.R-project.org/package=rematch2 |
| Dowle M, Srinivasan A. data.table: Extension of “data.frame” [Internet]. 2023. Available from: https://CRAN.R-project.org/package=data.table |
| Du Y, Varadhan R. SQUAREM:  An R package for off-the-shelf acceleration of EM, MM and other  EM-like monotone algorithms. Journal of Statistical Software. 2020;92(7):1–41. |
| Eddelbuettel D, Balamuta JJ. Extending  extitR with extitC++: A Brief Introduction to extitRcpp. The American Statistician. 2018;72(1):28–36. |
| Eddelbuettel D, Emerson JW, Kane MJ. BH: Boost c++ header files [Internet]. 2023. Available from: https://CRAN.R-project.org/package=BH |
| Eddelbuettel D, François R. Rcpp:  Seamless R and C++ integration. Journal of Statistical Software. 2011;40(8):1–18. |
| Eddelbuettel D. Seamless  R and C++ integration with Rcpp. New York: Springer; 2013. |
| FitzJohn R. ids: Generate random identifiers [Internet]. 2017. Available from: https://CRAN.R-project.org/package=ids |
| Fox J, Weisberg S, Price B. carData: Companion to applied regression data sets [Internet]. 2022. Available from: https://CRAN.R-project.org/package=carData |
| Fox J, Weisberg S. An R companion to applied regression [Internet]. Third. Thousand Oaks CA: Sage; 2019. Available from: https://socialsciences.mcmaster.ca/jfox/Books/Companion/ |
| Gagolewski M. stringi:  Fast and portable character string processing in R. Journal of Statistical Software. 2022;103(2):1–59. |
| Garbett SP, Stephens J, Simonov K, Xie Y, Dong Z, Wickham H, et al. yaml: Methods to convert r data to YAML and back [Internet]. 2023. Available from: https://CRAN.R-project.org/package=yaml |
| Garnier, Simon, Ross, Noam, Rudis, Robert, et al. viridis(Lite) - colorblind-friendly color maps for r [Internet]. 2023. Available from: https://sjmgarnier.github.io/viridis/ |
| Gaslam B. diffobj: Diffs for r objects [Internet]. 2021. Available from: https://CRAN.R-project.org/package=diffobj |
| Gaslam B. fansi: ANSI control sequence aware string functions [Internet]. 2023. Available from: https://CRAN.R-project.org/package=fansi |
| Genz A, Bretz F. Computation of multivariate normal and t probabilities. Heidelberg: Springer-Verlag; 2009. (Lecture notes in statistics). |
| Gerds TA. prodlim: Product-limit estimation for censored event history analysis [Internet]. 2023. Available from: https://CRAN.R-project.org/package=prodlim |
| Gilbert P, Varadhan R. numDeriv: Accurate numerical derivatives [Internet]. 2019. Available from: https://CRAN.R-project.org/package=numDeriv |
| Halekoh U, Højsgaard S. A kenward-roger approximation and parametric bootstrap methods for tests in linear mixed models – the R package pbkrtest. Journal of Statistical Software [Internet]. 2014;59(9):1–30. Available from: https://www.jstatsoft.org/v59/i09/ |
| Henry L, Wickham H. lifecycle: Manage the life cycle of your package functions [Internet]. 2022. Available from: https://CRAN.R-project.org/package=lifecycle |
| Henry L, Wickham H. tidyselect: Select from a set of strings [Internet]. 2022. Available from: https://CRAN.R-project.org/package=tidyselect |
| Hester J, Bryan J. glue: Interpreted string literals [Internet]. 2022. Available from: https://CRAN.R-project.org/package=glue |
| Hester J, Csárdi G. brio: Basic r input output [Internet]. 2021. Available from: https://CRAN.R-project.org/package=brio |
| Hester J, François R. cpp11: A c++11 interface for r’s c interface [Internet]. 2022. Available from: https://CRAN.R-project.org/package=cpp11 |
| Hester J, Henry L, Müller K, Ushey K, Wickham H, Chang W. withr: Run code “With” temporarily modified global state [Internet]. 2022. Available from: https://CRAN.R-project.org/package=withr |
| Hester J, Wickham H, Bryan J. vroom: Read and write rectangular text data quickly [Internet]. 2023. Available from: https://CRAN.R-project.org/package=vroom |
| Hester J, Wickham H, Csárdi G. fs: Cross-platform file system operations based on “libuv” [Internet]. 2023. Available from: https://CRAN.R-project.org/package=fs |
| Holst KK, Budtz-Joergensen E. A  two-stage estimation procedure for non-linear structural equation  models. Biostatistics. 2020;21(4):676–91. |
| Holst KK, Budtz-Joergensen E. Linear  latent variable models: The lava-package. Computational Statistics. 2013;28(4):1385–452. |
| Hosking JRM. L-moments [Internet]. 2022. Available from: https://CRAN.R-project.org/package=lmom |
| Hunt T. ModelMetrics: Rapid calculation of model metrics [Internet]. 2020. Available from: https://CRAN.R-project.org/package=ModelMetrics |
| Iannone R. fontawesome: Easily work with “Font Awesome” icons [Internet]. 2023. Available from: https://CRAN.R-project.org/package=fontawesome |
| Johnson SG. The NLopt nonlinear-optimization package. ? ?(?)? |
| Justin Talbot. labeling: Axis labeling [Internet]. 2020. Available from: https://CRAN.R-project.org/package=labeling |
| Kassambara A. ggpubr: “ggplot2” based publication ready plots [Internet]. 2023. Available from: https://CRAN.R-project.org/package=ggpubr |
| Kassambara A. rstatix: Pipe-friendly framework for basic statistical tests [Internet]. 2023. Available from: https://CRAN.R-project.org/package=rstatix |
| King R, Dean B, Klinke S, van Staden P. gld: Estimation and use of the generalised (tukey) lambda distribution [Internet]. 2022. Available from: https://CRAN.R-project.org/package=gld |
| Koenker R. quantreg: Quantile regression [Internet]. 2023. Available from: https://CRAN.R-project.org/package=quantreg |
| Koenker R. SparseM: Sparse linear algebra [Internet]. 2021. Available from: https://CRAN.R-project.org/package=SparseM |
| Kuhn M, Wickham H, Hvitfeldt E. recipes: Preprocessing and feature engineering steps for modeling [Internet]. 2023. Available from: https://CRAN.R-project.org/package=recipes |
| Kuhn, Max. Building predictive models in r using the caret package. Journal of Statistical Software [Internet]. 2008;28(5):1–26. Available from: https://www.jstatsoft.org/index.php/jss/article/view/v028i05 |
| Lang M, R Core Team. backports: Reimplementations of functions introduced since r-3.0.0 [Internet]. 2021. Available from: https://CRAN.R-project.org/package=backports |
| Lincoln M. clipr: Read and write from the system clipboard [Internet]. 2022. Available from: https://CRAN.R-project.org/package=clipr |
| Loden J, Daeschler D, Rodola’ G, Csárdi G. ps: List, query, manipulate system processes [Internet]. 2023. Available from: https://CRAN.R-project.org/package=ps |
| Lumley T. dichromat: Color schemes for dichromats [Internet]. 2022. Available from: https://CRAN.R-project.org/package=dichromat |
| Maechler M, Dutang C, Goulet V. expm: Matrix exponential, log, “etc” [Internet]. 2023. Available from: https://CRAN.R-project.org/package=expm |
| Meyer D, Buchta C. proxy: Distance and similarity measures [Internet]. 2022. Available from: https://CRAN.R-project.org/package=proxy |
| Meyer D, Dimitriadou E, Hornik K, Weingessel A, Leisch F. e1071: Misc functions of the department of statistics, probability theory group (formerly: E1071), TU wien [Internet]. 2023. Available from: https://CRAN.R-project.org/package=e1071 |
| Microsoft, Weston S. foreach: Provides foreach looping construct [Internet]. 2022. Available from: https://CRAN.R-project.org/package=foreach |
| Müller K. rprojroot: Finding files in project subdirectories [Internet]. 2022. Available from: https://CRAN.R-project.org/package=rprojroot |
| Neuwirth E. RColorBrewer: ColorBrewer palettes [Internet]. 2022. Available from: https://CRAN.R-project.org/package=RColorBrewer |
| Oehlschlägel J, Ripley B. bit: Classes and methods for fast memory-efficient boolean selections [Internet]. 2022. Available from: https://CRAN.R-project.org/package=bit |
| Oehlschlägel J, Silvestri L. bit64: A S3 class for vectors of 64bit integers [Internet]. 2020. Available from: https://CRAN.R-project.org/package=bit64 |
| Ooms J. askpass: Safe password entry for r, git, and SSH [Internet]. 2019. Available from: https://CRAN.R-project.org/package=askpass |
| Ooms J. curl: A modern and flexible web client for r [Internet]. 2023. Available from: https://CRAN.R-project.org/package=curl |
| Ooms J. openssl: Toolkit for encryption, signatures and certificates based on OpenSSL [Internet]. 2023. Available from: https://CRAN.R-project.org/package=openssl |
| Ooms J. sys: Powerful and reliable tools for running system commands in r [Internet]. 2023. Available from: https://CRAN.R-project.org/package=sys |
| Pedersen TL, Nicolae B, François R. farver: High performance colour space manipulation [Internet]. 2022. Available from: https://CRAN.R-project.org/package=farver |
| Pedersen TL, Ooms J, Govett D. systemfonts: System native font finding [Internet]. 2022. Available from: https://CRAN.R-project.org/package=systemfonts |
| Pedersen TL. textshaping: Bindings to the “HarfBuzz” and “Fribidi” libraries for text shaping [Internet]. 2021. Available from: https://CRAN.R-project.org/package=textshaping |
| Perry PO. utf8: Unicode text processing [Internet]. 2023. Available from: https://CRAN.R-project.org/package=utf8 |
| Peters A, Hothorn T. ipred: Improved predictors [Internet]. 2023. Available from: https://CRAN.R-project.org/package=ipred |
| Plate T, Heiberger R. abind: Combine multidimensional arrays [Internet]. 2016. Available from: https://CRAN.R-project.org/package=abind |
| Posit team. RStudio: Integrated development environment for r [Internet]. Boston, MA: Posit Software, PBC; 2023. Available from: http://www.posit.co/ |
| Potter S. Introducing the selectr package [Internet]. Auckland, New Zealand: The University of Auckland; 2012. Available from: http://stattech.wordpress.fos.auckland.ac.nz/2012-10-introducing-the-selectr-package/ |
| R by Ray Brownrigg DMcIlroyP for, Minka TP, Plan 9 codebase by Roger Bivand. transition to. mapproj: Map projections [Internet]. 2023. Available from: https://CRAN.R-project.org/package=mapproj |
| R Core Team. R: A language and environment for statistical computing [Internet]. Vienna, Austria: R Foundation for Statistical Computing; 2023. Available from: https://www.R-project.org/ |
| R Special Interest Group on Databases (R-SIG-DB), Wickham H, Müller K. DBI: R database interface [Internet]. 2022. Available from: https://CRAN.R-project.org/package=DBI |
| Ratnakumar S, Mick T, Davis T. rappdirs: Application directories: Determine where to save data, caches, and logs [Internet]. 2021. Available from: https://CRAN.R-project.org/package=rappdirs |
| Richard A. Becker OS code by, Ray Brownrigg. Enhancements by Thomas P Minka ARWilksR version by, Deckmyn. A. maps: Draw geographical maps [Internet]. 2022. Available from: https://CRAN.R-project.org/package=maps |
| Robin X, Turck N, Hainard A, Tiberti N, Lisacek F, Sanchez JC, et al. pROC: An open-source package for r and s+ to analyze and compare ROC curves. BMC Bioinformatics. 2011;12:77. |
| Sievert C, Cheng J. bslib: Custom “Bootstrap” “Sass” themes for “shiny” and “rmarkdown” [Internet]. 2022. Available from: https://CRAN.R-project.org/package=bslib |
| Sievert C, Cheng J. jquerylib: Obtain “jQuery” as an HTML dependency object [Internet]. 2021. Available from: https://CRAN.R-project.org/package=jquerylib |
| Signorell A. DescTools: Tools for descriptive statistics [Internet]. 2023. Available from: https://CRAN.R-project.org/package=DescTools |
| Slowikowski K. ggrepel: Automatically position non-overlapping text labels with “ggplot2” [Internet]. 2023. Available from: https://CRAN.R-project.org/package=ggrepel |
| Soetaert K, Herman PMJ. A practical guide to ecological modelling. Using r as a simulation platform. Springer; 2009. |
| Soetaert K. diagram: Functions for visualising simple graphs (networks), plotting flow diagrams [Internet]. 2020. Available from: https://CRAN.R-project.org/package=diagram |
| Soetaert K. rootSolve: Nonlinear root finding, equilibrium and steady-state analysis of ordinary differential equations. 2009. |
| Soetaert K. shape: Functions for plotting graphical shapes, colors [Internet]. 2021. Available from: https://CRAN.R-project.org/package=shape |
| Spinu V. timechange: Efficient manipulation of date-times [Internet]. 2023. Available from: https://CRAN.R-project.org/package=timechange |
| Stauffer R, Mayr GJ, Dabernig M, Zeileis A. Somewhere  over the rainbow: How to make effective use of colors in  meteorological visualizations. Bulletin of the American Meteorological Society. 2009;96(2):203–16. |
| Urbanek S, Ts’o T. uuid: Tools for generating and handling of UUIDs [Internet]. 2022. Available from: https://CRAN.R-project.org/package=uuid |
| Urbanek S. base64enc: Tools for base64 encoding [Internet]. 2015. Available from: https://CRAN.R-project.org/package=base64enc |
| Ushey K. renv: Project environments [Internet]. 2023. Available from: https://CRAN.R-project.org/package=renv |
| van der Loo M. gower: Gower’s distance [Internet]. 2022. Available from: https://CRAN.R-project.org/package=gower |
| Vaughan D, Kuhn M. hardhat: Construct modeling packages [Internet]. 2023. Available from: https://CRAN.R-project.org/package=hardhat |
| Vaughan D. clock: Date-time types and tools [Internet]. 2023. Available from: https://CRAN.R-project.org/package=clock |
| Vaughan D. tzdb: Time zone database information [Internet]. 2023. Available from: https://CRAN.R-project.org/package=tzdb |
| Venables B, Hornik K, Maechler M. polynom: A collection of functions to implement a class for univariate polynomial manipulations [Internet]. 2022. Available from: https://CRAN.R-project.org/package=polynom |
| Wei T, Simko V. R package “corrplot”: Visualization of a correlation matrix [Internet]. 2021. Available from: https://github.com/taiyun/corrplot |
| Wickham C. munsell: Utilities for using munsell colours [Internet]. 2018. Available from: https://CRAN.R-project.org/package=munsell |
| Wickham H, Averick M, Bryan J, Chang W, McGowan LD, François R, et al. Welcome to the  tidyverse. Journal of Open Source Software. 2019;4(43):1686. |
| Wickham H, Chang W, Hester J, Henry L. pkgload: Simulate package installation and attach [Internet]. 2022. Available from: https://CRAN.R-project.org/package=pkgload |
| Wickham H, Henry L, Pedersen TL, Luciani TJ, Decorde M, Lise V. svglite: An “SVG” graphics device [Internet]. 2023. Available from: https://CRAN.R-project.org/package=svglite |
| Wickham H, Henry L, Vaughan D. vctrs: Vector helpers [Internet]. 2023. Available from: https://CRAN.R-project.org/package=vctrs |
| Wickham H, Hester J, Chang W, Müller K, Cook D. memoise: “Memoisation” of functions [Internet]. 2021. Available from: https://CRAN.R-project.org/package=memoise |
| Wickham H, Kuhn M, Vaughan D. generics: Common S3 generics not provided by base r methods related to model fitting [Internet]. 2022. Available from: https://CRAN.R-project.org/package=generics |
| Wickham H, Pedersen TL. gtable: Arrange “Grobs” in tables [Internet]. 2023. Available from: https://CRAN.R-project.org/package=gtable |
| Wickham H, Seidel D. scales: Scale functions for visualization [Internet]. 2022. Available from: https://CRAN.R-project.org/package=scales |
| Wickham H, Wilke CO, Pedersen TL. isoband: Generate isolines and isobands from regularly spaced elevation grids [Internet]. 2022. Available from: https://CRAN.R-project.org/package=isoband |
| Wickham H, Xie Y. evaluate: Parsing and evaluation tools that provide more details than the default [Internet]. 2023. Available from: https://CRAN.R-project.org/package=evaluate |
| Wickham H. blob: A simple S3 class for representing vectors of binary data (“BLOBS”) [Internet]. 2023. Available from: https://CRAN.R-project.org/package=blob |
| Wickham H. ellipsis: Tools for working with ... [Internet]. 2021. Available from: https://CRAN.R-project.org/package=ellipsis |
| Wickham H. Reshaping data with the reshape package. Journal of Statistical Software [Internet]. 2007;21(12):1–20. Available from: http://www.jstatsoft.org/v21/i12/ |
| Wickham H. testthat: Get started with testing. The R Journal [Internet]. 2011;3:5–10. Available from: https://journal.r-project.org/archive/2011-1/RJournal_2011-1_Wickham.pdf |
| Wickham H. The split-apply-combine strategy for data analysis. Journal of Statistical Software [Internet]. 2011;40(1):1–29. Available from: https://www.jstatsoft.org/v40/i01/ |
| Wickham H. waldo: Find differences between r objects [Internet]. 2023. Available from: https://CRAN.R-project.org/package=waldo |
| Wilke CO. cowplot: Streamlined plot theme and plot annotations for “ggplot2” [Internet]. 2020. Available from: https://CRAN.R-project.org/package=cowplot |
| Wright K. pals: Color palettes, colormaps, and tools to evaluate them [Internet]. 2021. Available from: https://CRAN.R-project.org/package=pals |
| Wuertz D, Setz T, Chalabi Y, Boshnakov GN. timeDate: Rmetrics - chronological and calendar objects [Internet]. 2023. Available from: https://CRAN.R-project.org/package=timeDate |
| Xiao N. ggsci: Scientific journal and sci-fi themed color palettes for “ggplot2” [Internet]. 2023. Available from: https://CRAN.R-project.org/package=ggsci |
| Xie Y, Allaire JJ, Grolemund G. R markdown: The definitive guide [Internet]. Boca Raton, Florida: Chapman; Hall/CRC; 2018. Available from: https://bookdown.org/yihui/rmarkdown |
| Xie Y, Dervieux C, Riederer E. R markdown cookbook [Internet]. Boca Raton, Florida: Chapman; Hall/CRC; 2020. Available from: https://bookdown.org/yihui/rmarkdown-cookbook |
| Xie Y, Qiu Y. highr: Syntax highlighting for r source code [Internet]. 2022. Available from: https://CRAN.R-project.org/package=highr |
| Xie Y. Dynamic documents with R and knitr [Internet]. 2nd ed. Boca Raton, Florida: Chapman; Hall/CRC; 2015. Available from: https://yihui.org/knitr/ |
| Xie Y. knitr: A comprehensive tool for reproducible research in R. In: Stodden V, Leisch F, Peng RD, editors. Implementing reproducible computational research. Chapman; Hall/CRC; 2014. |
| Xie Y. knitr: A general-purpose package for dynamic report generation in r [Internet]. 2023. Available from: https://yihui.org/knitr/ |
| Xie Y. mime: Map filenames to MIME types [Internet]. 2021. Available from: https://CRAN.R-project.org/package=mime |
| Xie Y. TinyTeX: A lightweight, cross-platform, and easy-to-maintain LaTeX distribution based on TeX live. TUGboat [Internet]. 2019;40(1):30–2. Available from: https://tug.org/TUGboat/Contents/contents40-1.html |
| Xie Y. tinytex: Helper functions to install and maintain TeX live, and compile LaTeX documents [Internet]. 2023. Available from: https://github.com/rstudio/tinytex |
| Xie Y. xfun: Supporting functions for packages maintained by “Yihui Xie” [Internet]. 2023. Available from: https://CRAN.R-project.org/package=xfun |
| Zeileis A, Fisher JC, Hornik K, Ihaka R, McWhite CD, Murrell P, et al. colorspace: A toolbox  for manipulating and assessing colors and palettes. Journal of Statistical Software. 2020;96(1):1–49. |
| Zeileis A, Hornik K, Murrell P. Escaping  RGBland: Selecting colors for statistical graphics. Computational Statistics & Data Analysis. 2009;53(9):3259–70. |
| Zhu H. kableExtra: Construct complex table with “kable” and pipe syntax [Internet]. 2021. Available from: https://CRAN.R-project.org/package=kableExtra |
